# sEMG dataset of simulated pain-related behaviours for robotic orthopaedic shoulder rehabilitation

**DOI:** 10.1016/j.dib.2026.113312

**Published:** 2026-10-05

**Authors:** Giulio Besi, Alessandra Fava, Haoxiang Wu, Sara Garavini, Lorenzo Sabattini, Federica Ferraguti

**Affiliations:** a Department of Sciences and Methods for Engineering, University of Modena and Reggio Emilia, via Giovanni Amendola, 2, Reggio Emilia, RE, 42122, Italy; b Department of Engineering for Innovation Medicine, University of Verona, Strada le Grazie, 15, Verona, VR, 37134, Italy; c Studio Canali: Physiotherapy, Osteopathy and Personal Training, Via Bologna, 6, Reggio Emilia, RE, 42123, Italy

**Keywords:** Robotic manipulator, Orthopaedic rehabilitation, Machine learning for rehabilitation, muscular activity analysis

## Abstract

This dataset contains surface electromyography (sEMG) recordings from 25 healthy participants aged 21–41 years performing shoulder abduction, shoulder flexion, and scapular retraction (“rower”) with a robotic manipulator. Upon oral cues, participants simulated three pain-related behaviours observed during shoulder rehabilitation and selected in consultation with an expert physiotherapist. Signals were recorded from the anterior and rear deltoids and the upper and lower trapezius using a Trigno Centro system and four wireless, single-channel Avanti sensors (Delsys Europe, Greater Manchester, UK).

To the best of the authors’ knowledge, frequently used pain-detection datasets contain signals recorded during externally induced pain. However, during real orthopaedic rehabilitation sessions, pain is never voluntarily and externally induced. Pain during movement can alter muscle activation and motor capacity, while measures of muscle activity, such as the nociceptive flexion reflex (NFR), are used in pain assessment and are normally measured using sEMG signals. This motivated the use of sEMG alone. The aim of this dataset is to provide a simple yet efficient source for designing machine learning algorithms for pain detection. Moreover, to the best of the authors’ knowledge, no currently publicly available dataset contains signals recorded during rehabilitation exercises performed using a robot.

Specifications Table

**Table:** 

Subject	Health Sciences, Medical Sciences & Pharmacology
Specific subject area	Human-robot interaction, Physiological signal analysis, Robotic upper-limb rehabilitation, Orthopaedic shoulder rehabilitation
Type of data	Data format: Raw, Filtered, Processed Type of data: EMG signal, Pain Labels, EMG reference values
Data collection	sEMG signals were recorded in a controlled laboratory from healthy participants performing three orthopaedic shoulder rehabilitation exercises. Participants simulated pain-related behaviours when prompted. Signals were acquired using a Trigno Centro system and four wireless, single-channel Avanti EMG sensors (Delsys Europe), positioned over the anterior and rear deltoids and the upper and lower trapezius. A physiotherapy expert defined the exercises, sensor positions, and simulated behaviours. A custom Python application was used to coordinate data collection. Informed consent was obtained, and subjects with musculoskeletal disorders were excluded.
Data Source Location	ARSControl Lab, Department of Sciences and Methods for Engineering, University of Modena and Reggio Emilia, Piazzale Europa 1, 42,124, Reggio Emilia, RE, Italy
Data accessibility	Repository name: Simulated Pain in Robotic Orthopaedic Shoulder Rehabilitation Data identification number: 10.5281/zenodo.21573324 Direct URL to data: https://zenodo.org/records/21573324 Instructions for accessing these data: visit the URL of the dataset, download the .zip file and extract the content.
Related research article	None

## 1 Value of the Data

• **First dataset for detecting shoulder pain during movement**: the data support the development of algorithms for detecting pain-related behaviours during orthopaedic shoulder exercises performed with a robotic manipulator. To the best of the authors’ knowledge, this is the first publicly available dataset designed for this specific setting.
• **Robotic orthopaedic rehabilitation vs neurorehabilitation**: research on robotic upper-limb rehabilitation has largely focused on neurorehabilitation, such as post-stroke recovery, while orthopaedic rehabilitation has received less attention. The absence of a dataset addressing pain-related behaviours in robotic orthopaedic shoulder rehabilitation motivated this work.
• **Rehabilitation exercise specificity**: recordings were made during shoulder abduction, flexion, and rower exercises while participants interacted with a robotic manipulator. They therefore capture muscle activity during exercises relevant to robot-assisted rehabilitation, rather than relying solely on externally induced pain.
• **Data subjectivity reduction**: sEMG is widely used for biofeedback in rehabilitation [1] and records muscle activity relevant to pain-assessment measures such as the NFR. Focusing on sEMG limits reliance on other physiological measures, including EDA and HRV, which may be consciously modulated [2,3]. The recorded behaviours were nevertheless voluntarily simulated and do not represent true nociceptive responses. Nonetheless, by focusing exclusively on sEMG signals, the dataset aims to reduce subjectivity associated with biosignal selection while acknowledging the voluntary component of the simulated behaviours.
• **Machine learning readiness**: the dataset includes recordings at rest and during three exercises, each performed under two conditions: 10 repetitions without a time limit and 30 s without a repetition limit. These conditions provide different recording contexts for developing and evaluating automated detection algorithms.
• **Easy setup and data scalability**: the use of sEMG signals alone facilitates the replication of the experimental setup by reducing the total number of sensors required. It also supports straightforward data scalability, as additional muscles or signal types can be incorporated into the dataset by simply replicating the data-acquisition protocol.

## 2 Background

Robots show promise for repetitive, labour-intensive upper-limb rehabilitation tasks [4,5]. Robotic manipulators have attracted interest because their control capabilities offer greater versatility than exoskeletons [[6], [7], [8]]. However, their integration into rehabilitation centres has focused mainly on neurorehabilitation, such as post-stroke rehabilitation, while orthopaedic rehabilitation remains underexplored [9]. Addressing this shortage and integrating pain status into robotic control in this scenario is therefore an important research direction for improving patient safety.

Existing pain-detection datasets have supported the development of machine-learning algorithms [[10], [11], [12], [13], [14]]. However, to the best of the authors’ knowledge, none includes pain-related movements or behaviours recorded during robotic orthopaedic rehabilitation. Most datasets rely on artificially induced pain, which does not reflect realistic orthopaedic scenarios [[15], [16]]. Only [12] reports naturally occurring pain during movement, but its sparse and heterogeneous observer labels limit its suitability for training automated detection models; moreover, its recordings were not obtained during robot-assisted rehabilitation. Therefore, the present dataset was created to provide an initial source of simulated pain-related behaviours in robotic orthopaedic upper-limb rehabilitation.

## 3 Data Description

The dataset contains electromyography (EMG) recordings acquired while participants performed three exercises for orthopaedic shoulder rehabilitation using a robotic manipulator and simulated pain-related behaviours when prompted. In total, 25 healthy participants without reported motor impairments contributed to the dataset. The data for each participant were anonymised so that individual participants cannot be identified.

Each participant completed a single experimental session. Oral instructions regarding the exercise modalities were provided to the participants, while a researcher used a custom Python interface to monitor the muscular activity and label the signals. Each exercise was performed under two different protocols. In the first protocol, participants performed 10 repetitions without any time constraint. In the second, they performed the exercise for 30 s without any constraint on the number of repetitions. Before recording the exercises, maximum voluntary contraction (MVC) procedures were performed for all four monitored muscles.

Each MVC cycle consisted of a 30-s rest period followed by a 5-s maximum isometric contraction. This cycle was repeated three times, resulting in a total recording duration of 105 s. The rest periods, including the first one, were included to reduce the effects of muscle fatigue. The final MVC value for each muscle was calculated as the mean of the root mean square (RMS) values obtained from the three contractions. For each 5-s contraction, the RMS was calculated using a 500-ms moving window with a one-sample step. The moving RMS was defined as:

(1) $$X_{RMS}\left[i\right]=\sqrt{\frac{1}{N}\sum_{k=i-N+1}^{i}x^{2}\left[k\right]}$$

where x denotes the signal within the moving window, i is the window position, and N is the number of samples per window. Eq. (1) was applied separately to each 5-s contraction, and only the maximum RMS value was retained for each contraction, denoted as $X_{RMS}^{max}$. The final MVC RMS value was then calculated as:

(2) $$MVC_{RMS}=\frac{1}{3}\sum_{j=1}^{3}X_{RMS_{j}}^{max}$$

A detailed description and illustration of the isometric contractions are provided in the EXPERIMENTAL DESIGN, MATERIALS AND METHODS section.

The dataset is organized into a main directory containing two PDF documents and two subdirectories, called *Raw_Data* and *Final_Dataset* (Fig. 1). The PDF documents are:

• *sensor_placement_and_mvc.pdf*, which describes the precise steps for correctly placing the sensors and performing the MVC procedures – the document’s content and description can be found in section EXPERIMENTAL DESIGN, MATERIALS AND METHODS and Fig. 11.
• *informed_consent.pdf*, which contains the template for the informed consent form signed by participants to authorize data collection.

**Fig. 1:**
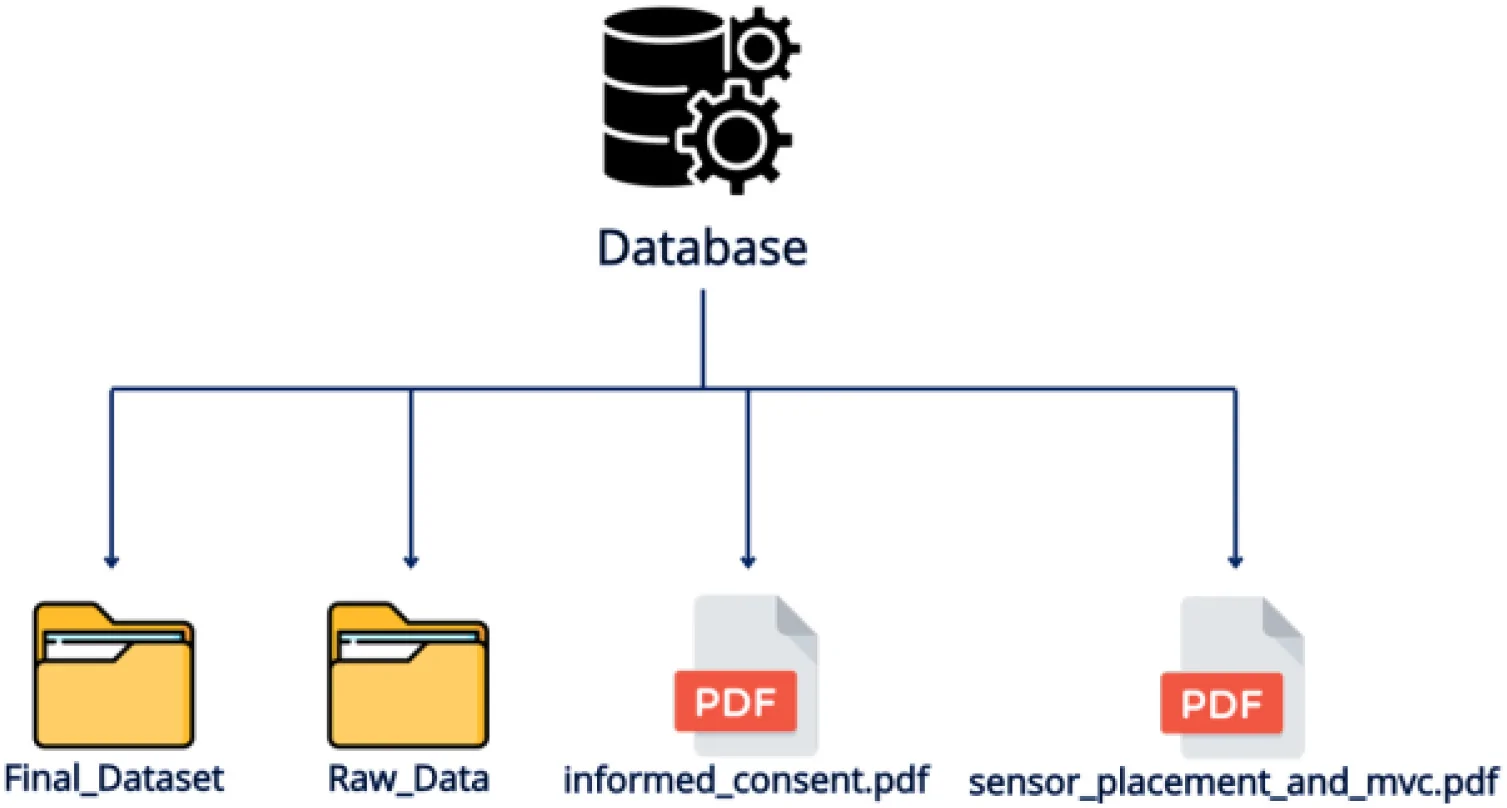
Main folder structure.

The contents and structure of the *Raw_Data* and *Final_Dataset* folders are described in the following subsections.

### 3.1 Raw_Data folder

*Raw_Data* contains one folder for each participant, identified by the prefix *Subject_* followed by a unique identification number (e.g. *Subject_07*). Each participant folder contains 18 CSV files divided into three categories: data (7 files), key (7 files), and MVC (4 files). The hierarchical structure of each folder can be seen in Fig. 2.

**Fig. 2:**
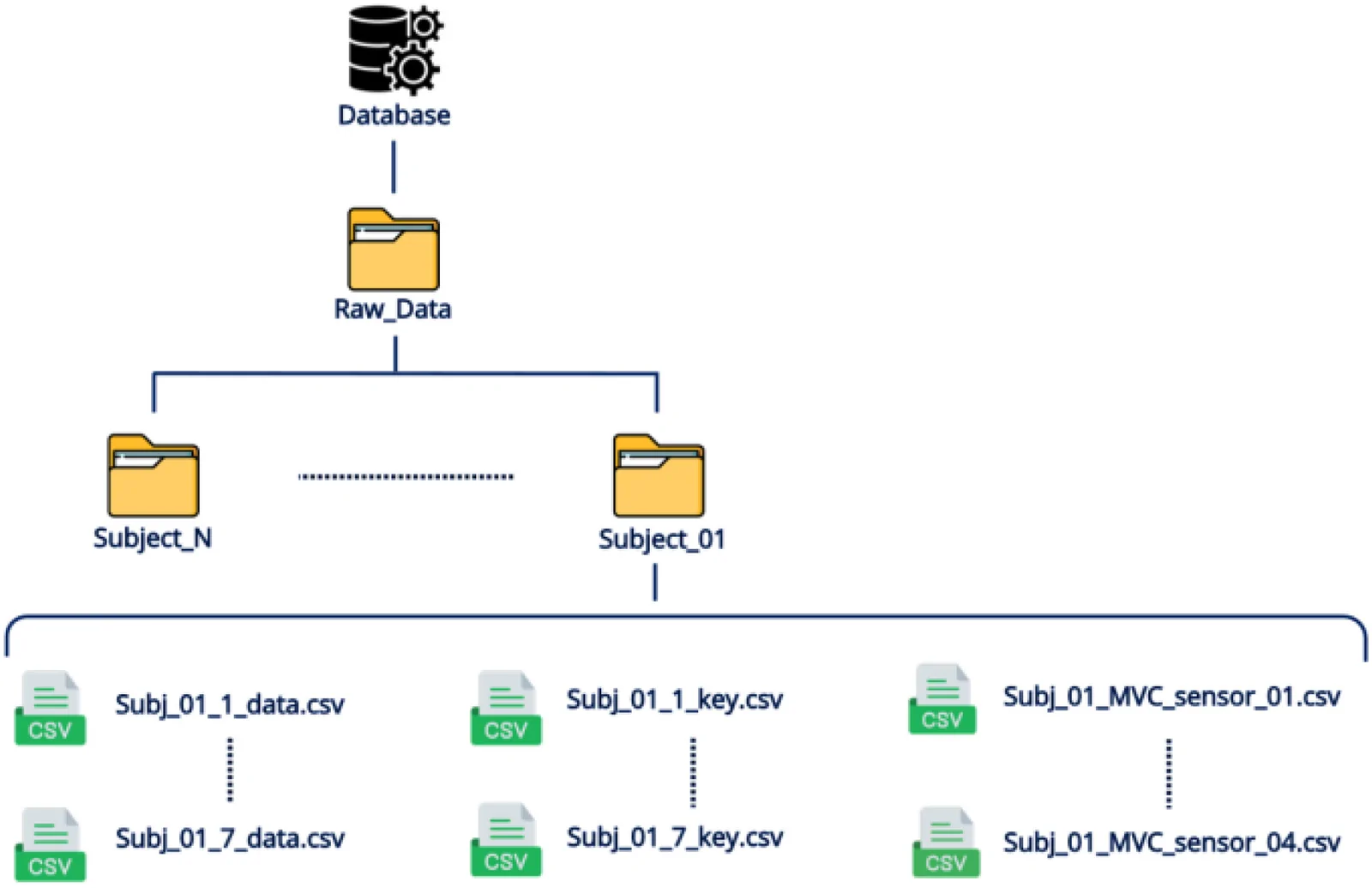
Raw_Data folder structure.

Excluding the MVC data, the data and key files are named *Subj_<subject_ID>_<exercise_ID>_data.csv* and *Subj_<subject_ID>_<exercise_ID>_key.csv*, respectively. Here, *exercise_ID* is a number indicating the recording phase stored in the file. The phases are numbered as follows:

1. Rest
2. Abduction – 10 repetitions
3. Abduction – 30 s
4. Flexion – 10 repetitions
5. Flexion – 30 s
6. Rower – 10 repetitions
7. Rower – 30 s

For example, *Subj_03_2_data.csv* and *Subj_03_2_key.csv* contain the data and key files, respectively, for subject 03 and recording phase 2. Further details on the individual phases are provided in the EXPERIMENTAL DESIGN, MATERIALS AND METHODS section. Each csv file contains:

1. the recording start time (first line);
2. a mapping between sensor IDs (i.e. sensor numbers) and muscles, which depends on the sensor placement (e.g., Sensor 01 → anterior deltoid, Sensor 02 → rear deltoid, Sensor 03 → upper trapezius, Sensor 04 → lower trapezius);
3. the recorded EMG signals, stored in three columns:
  *muscle_name*, indicating the corresponding muscle;
  *time_point*, containing the timestamp of each EMG data point;
  *value*, containing the one-dimensional EMG signal value.

Each key file contains the labels for the corresponding data file, identified by the same suffix, and is used to annotate the beginning and end of the simulated pain-related behaviour. Each key file is organised into two columns:

• *press_time*, indicating the time at which the pain simulation button was pressed;
• *release_time*, indicating the time at which the button was released.

Similarly, MVC files follow the naming convention *Subj_<subject_ID>_MVC_sensor_<sensor_ID>.csv* (e.g. Subj_01_MVC_sensor_01.csv). Each *MVC* file contains:

1. the recording start time (first line);
2. the sensor-to-muscle mapping for the sensor indicated in the filename and used during the recording (e.g., Sensor 01 → anterior deltoid);
3. the recorded MVC signal, stored in three columns with the same structure as the data files (i.e. *muscle_name, time_point*, and *value*).

Specific information on the MVC recording procedure is provided in the EXPERIMENTAL DESIGN, MATERIALS AND METHODS section.

### 3.2 Final_Dataset folder

As for the *Raw_Data* folder, the subdirectories corresponding to all recorded participants are contained within *Final_Dataset* and are uniquely identified by the subject ID. Each subject directory contains four folders: one containing data recorded during the rest phase and the remaining three containing data recorded during the exercise phases, namely abduction, flexion, and rower. These directories are created by processing the data from the corresponding subject folder in *Raw_Data*, as shown in Fig. 3.

**Fig. 3:**
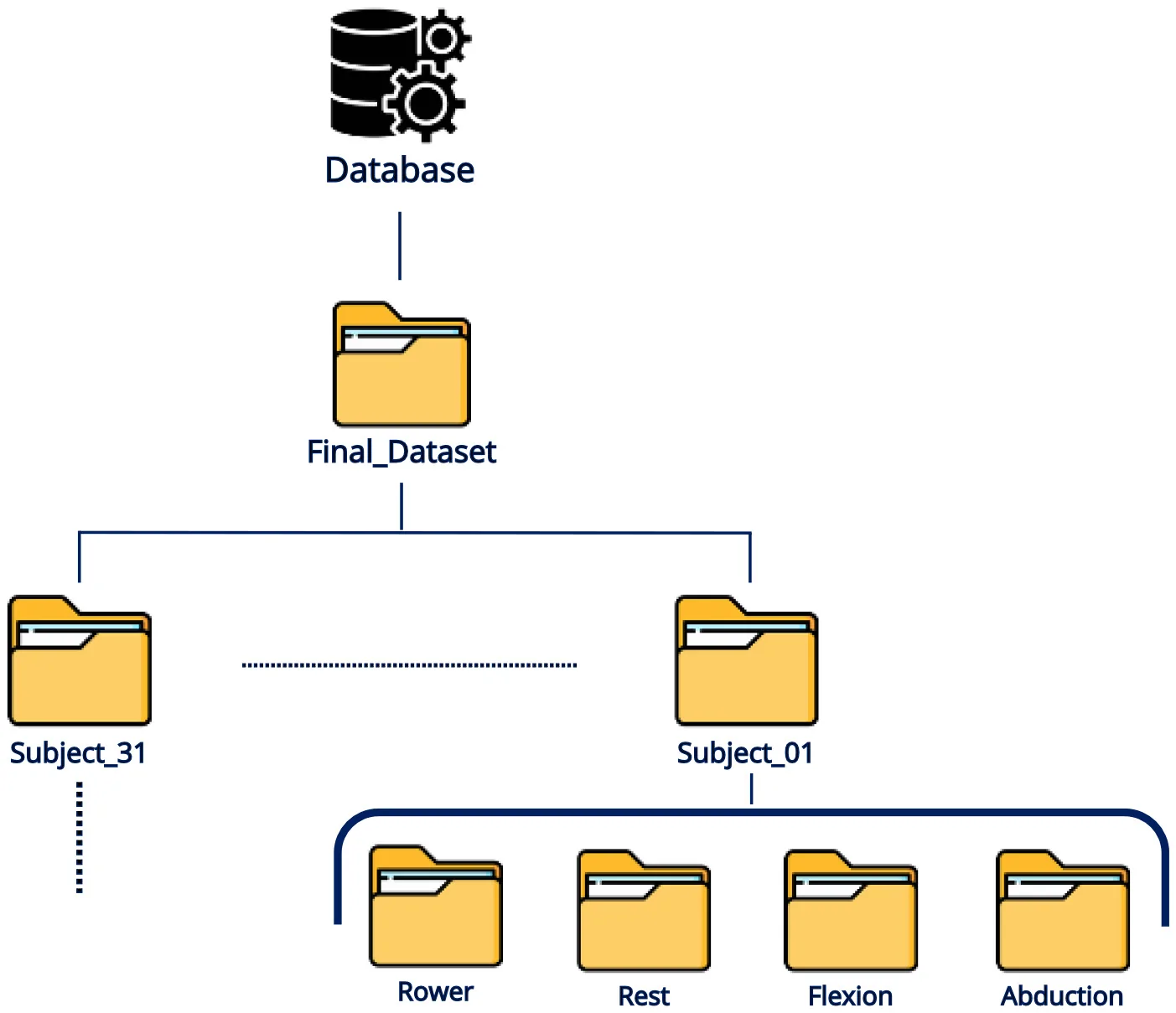
Final_Dataset folder structure (down to exercises level).

As shown in Fig. 4, each exercise folder is further divided into:

• *10_reps*, containing data recorded during the 10-repetition protocol
• *30_secs*, containing data recorded during the 30-second protocol
• *Sensor_to_muscle.txt*, containing the mapping between the Avanti sensor numbers and the muscles on which they were placed.

**Fig. 4:**
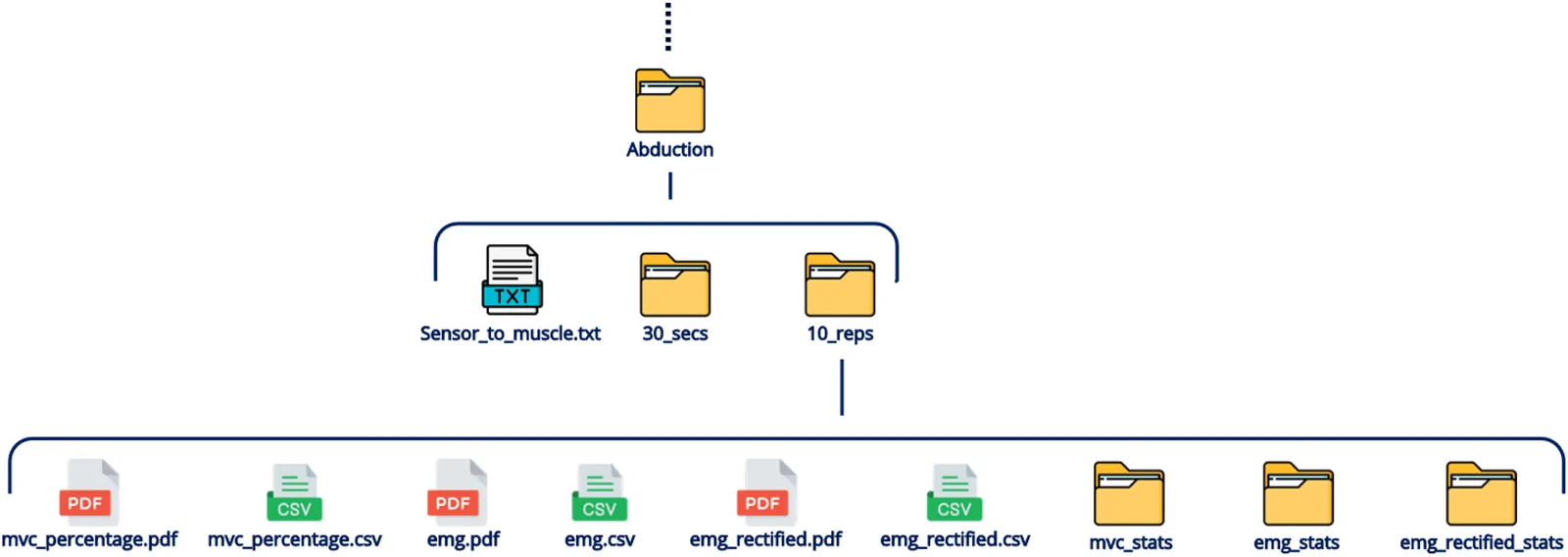
Example structure of an exercise folder (in this case, abduction).

Both the *10_reps* and *30_secs* folders contain the corresponding participant’s data, reorganised and processed into different formats, as shown in Fig. 4. Specifically, each protocol folder contains the following:

• *emg.csv*: a CSV file containing the raw data from *Raw_Data*, reorganised into six columns: one timestamp column (*time_point*), four columns for the recorded muscles (*Anterior_Deltoid, Lower_Trapezius, Rear_Deltoid*, and *Upper_Trapezius*), and one column for the binary pain labels (*Label*). Timestamp, muscle, and label values are expressed in seconds, millivolts, and scalar values, respectively.
• *emg.pdf*: a PDF file showing the temporal evolution of the signals contained in the corresponding CSV file, together with visual information about the assigned labels, represented by red areas in the plot.
• *emg_rectified.csv*: csv file with same data and format of *emg.csv*, but with rectified EMG values.
• *emg_rectified.pdf*: a PDF file showing the rectified EMG signals and the assigned labels, following the same format as *emg.pdf*.
• *mvc_percentage.csv*: a CSV file with the same columns as *emg.csv*, containing muscle values normalised using the MVC RMS values and expressed as percentages.
• *mvc_percentage.pdf*: a PDF file following the same format as the other PDF files.
• *emg_rectified_stats*: a folder containing text files with statistical values calculated from *emg_rectified.csv*.
• *emg_stats*: a folder containing text files with statistical values calculated from *emg.csv*.
• *mvc_stats*: a folder containing text files with statistical values calculated from the MVC signal files in *Raw_Data*.

As shown in Fig. 5, each _*stats* folder contains two text files for each Avanti sensor, providing information about the corresponding recording phase:

• *Sensor_<sensor_ID>_maxval.txt*, containing the maximum EMG value of the recorded signal in millivolts;
• *Sensor_<sensor_ID>_mean.txt*, containing the mean value of the recorded EMG signal.

**Fig. 5:**
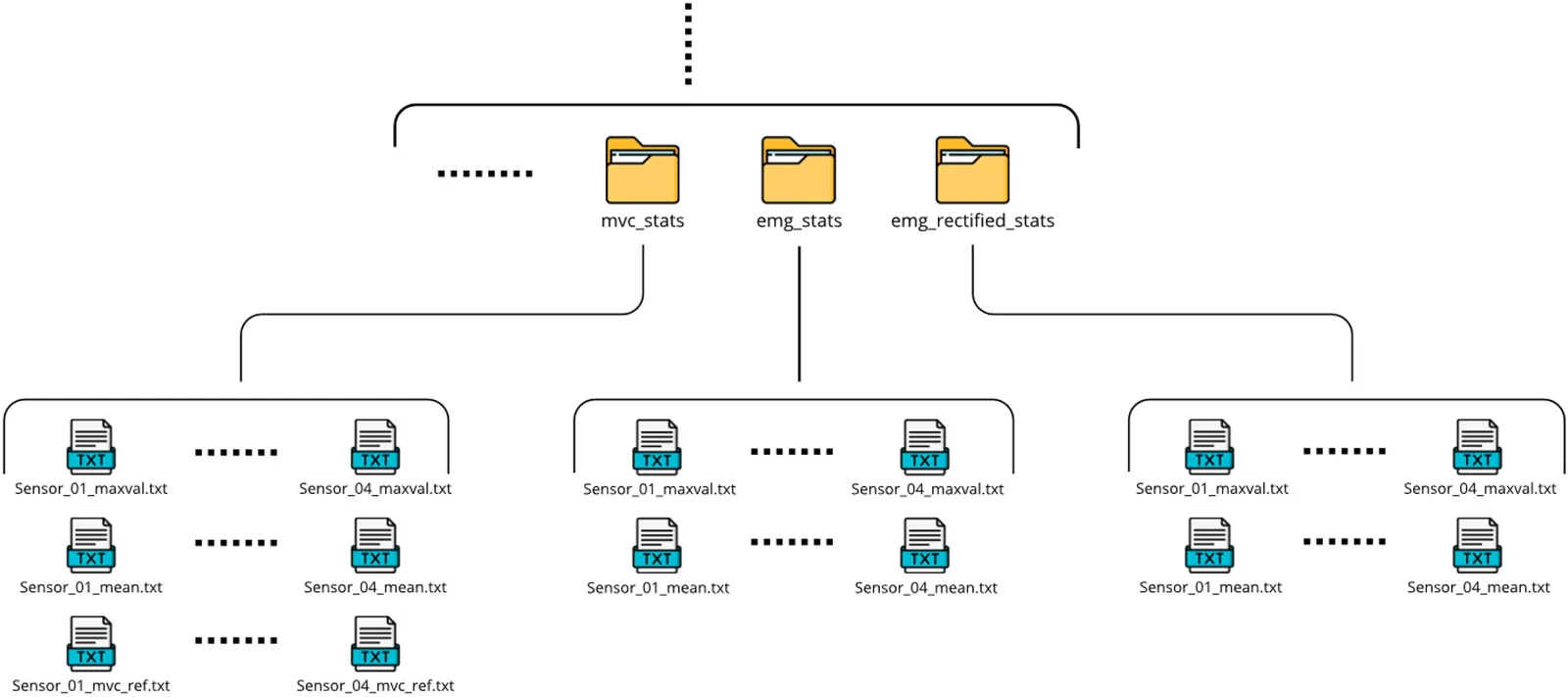
``_stats'' folders structure.

In addition to these two files, the *mvc_stats* folder contains a third text file for each sensor, namely *Sensor_<sensor_ID>_mvc_ref.txt*. This file contains the RMS value of the MVC signal, calculated as the mean of the maximum moving RMS values obtained from the three 5-second contraction windows. These values are used to normalise the signals in *mvc_percentage.csv* and are displayed in the legends of the plots contained in *mvc_percentage.pdf*.

The *Rest* folder follows the same directory structure as the *10_reps* folders. In addition, each exercise folder contains a *Sensor_to_muscle.txt* file alongside the *10_reps* and *30_secs* folders. This file specifies the mapping between each Avanti sensor and the muscle on which it was placed.

The data structure has been designed to enable users to navigate the dataset easily, isolate specific recordings, and reproduce the experimental procedure. The supporting documents facilitate replication of the sensor setup and provide the ethical framework for data collection. Moreover, the structure allows additional sensor data to be integrated, thereby supporting straightforward dataset expansion. Fig. 6 and Table 1 provide the mean and standard deviation of the sEMG signals and the label distributions, respectively, for each exercise.

**Fig. 6:**
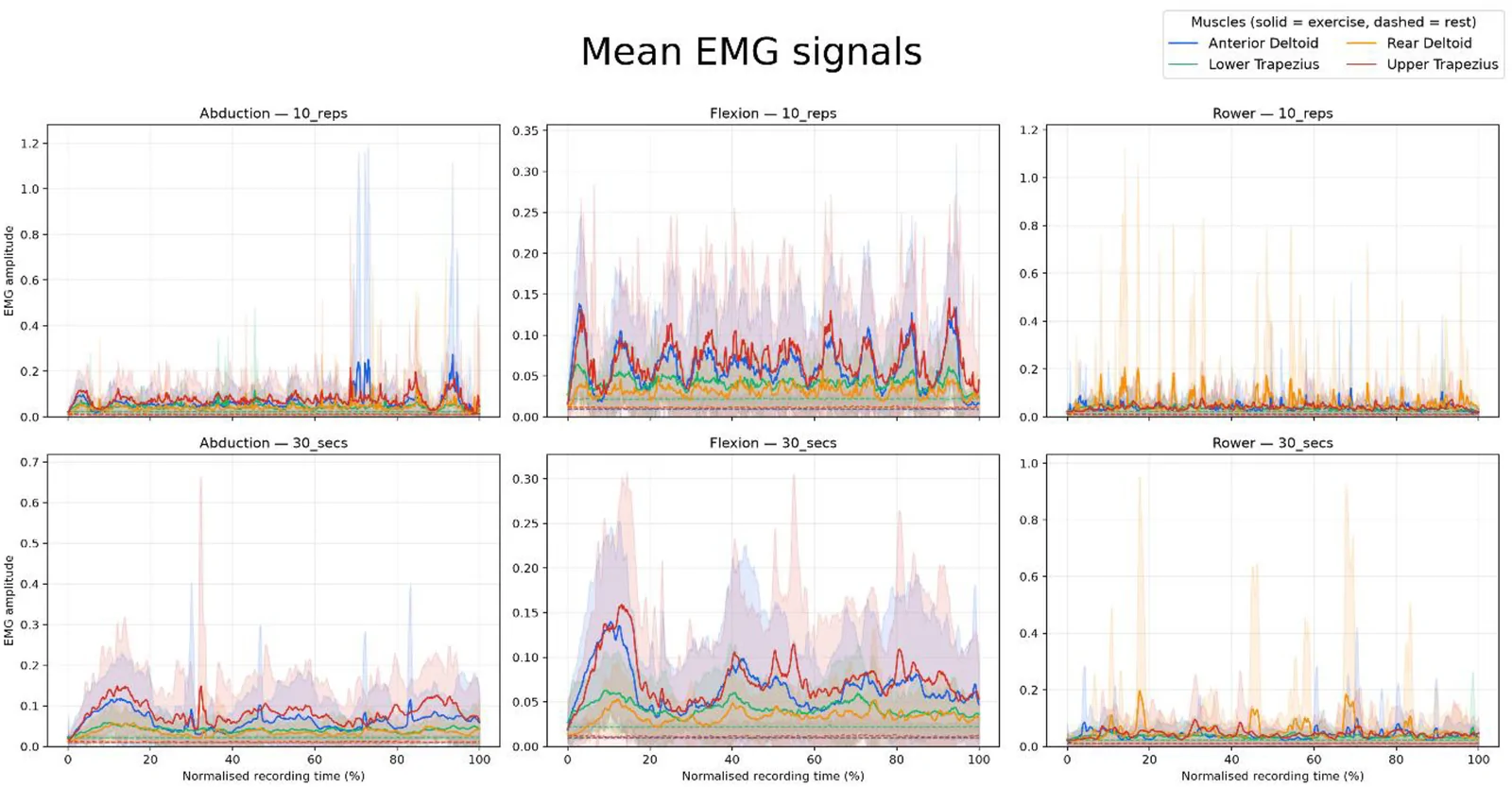
Mean sEMG signals recorded during the three exercises under both recording protocols (10 repetitions and 30 s). Solid lines represent the mean sEMG amplitude for each muscle across all 25 subjects, with shaded areas of the corresponding colour indicating ±1 standard deviation. Dashed lines represent the mean sEMG amplitude recorded at rest across all 25 subjects. The colours used for anterior deltoid, rear deltoid, lower trapezius and upper trapezius are blue, orange, green and red, respectively.

**Table 1:** label distribution across exercises.

Exercise	# recordings	Label 0 total time [s] (%)	Label 1 total time [s] (%)
Abduction	50	3744 (91.79)	335 (8.21)
Flexion	50	3409 (91.41)	320 (8.59)
Rower	50	2434 (89.87)	274 (10.13)

## 4 Experimental Design, Materials and Methods

### 4.1 Participants

A total of 25 healthy adult volunteers (15 males and 10 females), aged 21–41 years (mean age = 28.0 ±3.67 years), were recruited for this study. No requirement was imposed regarding the participants’ dominant hand, although all participants were right-handed. None reported a history of musculoskeletal disorders or presented any upper-limb impairment that could affect task execution. To ensure homogeneous data acquisition, participants were asked not to perform any physical activity on the day of the recording session. This measure was taken to minimise sources of muscle fatigue or reduced arousal that could interfere with muscular activity. Participants with considerable hair around the shoulder or beneath the scapula were advised to arrive with these regions shaved or groomed on the day of the experiment to maximise the recording capability of the EMG sensors. Although this was not mandatory, all participants complied with the request. Data were acquired in a controlled laboratory environment.

Before the recording session began, each participant was informed in detail about the experimental procedure and the type of data to be collected. Written informed consent was obtained from all participants, who also received a copy of the signed document. The study was conducted in accordance with the Declaration of Helsinki, and the experimental procedure was reviewed and approved by the Ethics Committee of the University of Modena and Reggio Emilia (Reggio Emilia, Emilia-Romagna, Italy) under protocol number 321,787/2025.

### 4.2 Instrumentation and sensor placement

Electromyographic activity was recorded using a Trigno Centro recording system (Delsys Europe) while participants performed shoulder-rehabilitation exercises by moving a UR10e robot (Universal Robots Inc., Michigan, USA). The Trigno Centro system comprises a radio station and four Avanti sensors, which were placed on the participants’ skin. Each Avanti sensor is a wireless module containing a single-channel EMG recording unit. The Avanti sensor setup requires users to select preset values for the sensor parameters. For EMG acquisition, the following settings were selected:

• Sampling frequency: 4370 Hz
• Voltage amplitude reading range: ±5.5 mV
• Butterworth band-pass filter: 10–850 Hz

It should be noted that the band-pass filter could not be deactivated because the Trigno Centro system does not provide this option. Therefore, the data described in Section DATA DESCRIPTION were hardware-filtered using the specified band-pass filter.

Each sensor was placed over one of the muscles commonly targeted and monitored during orthopaedic shoulder-rehabilitation exercises: the anterior deltoid, rear deltoid, upper trapezius, and lower trapezius. Before placement, the participants’ skin was cleaned to improve signal quality, facilitate sensor placement, and ensure that the sensors remained securely attached during limb movement. The sensors were then placed as shown in Fig. 7, Fig. 8, Fig. 9, Fig. 10, all by following the instructions provided in *sensor_placement_and_mvc.pdf* (Fig. 11).

**Fig. 7:**
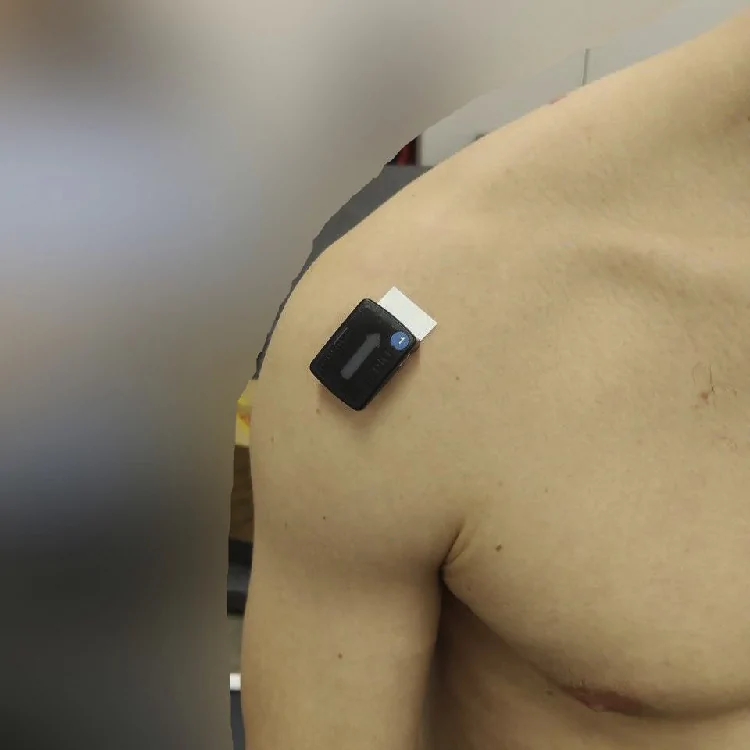
Sensor placement - anterior deltoid.

**Fig. 8:**
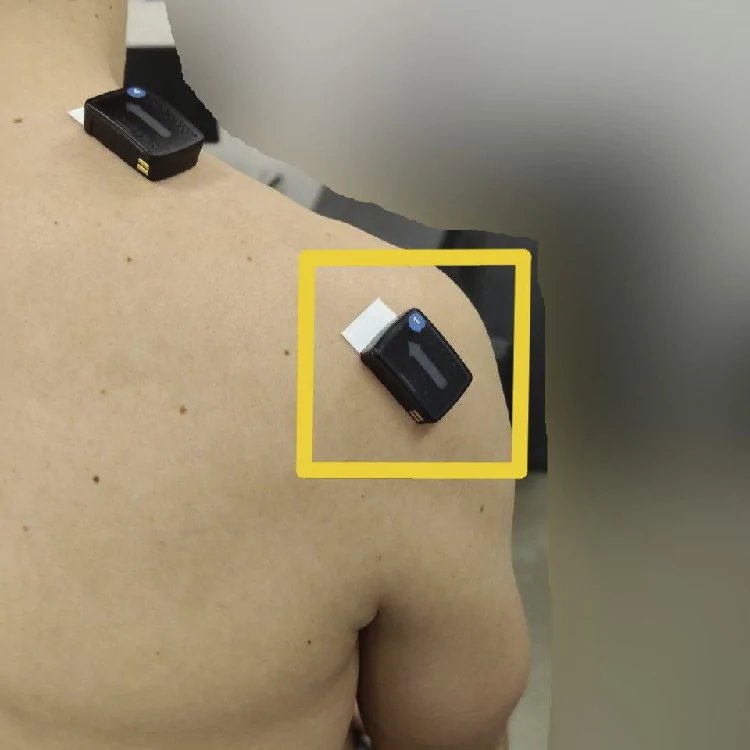
Sensor placement - rear deltoid.

**Fig. 9:**
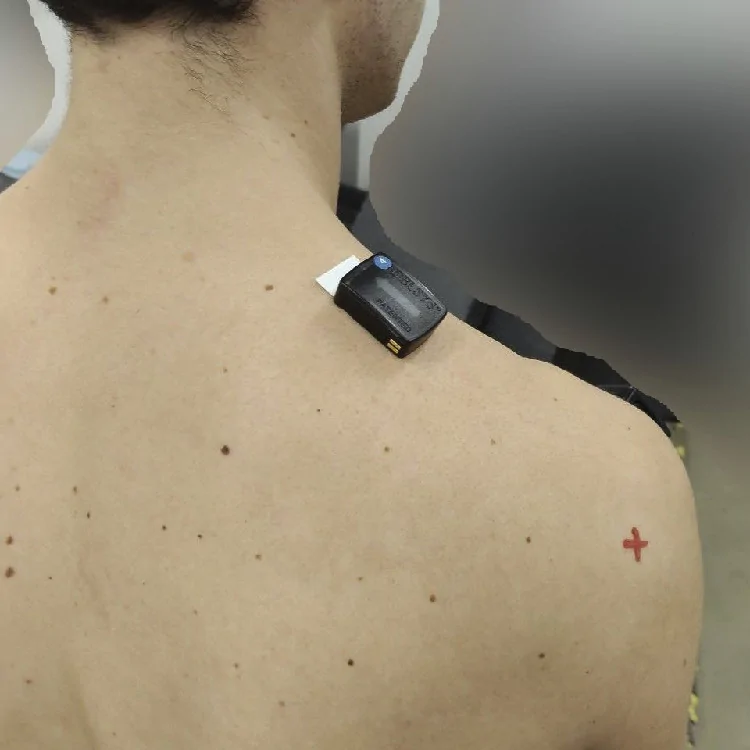
Sensor placement - upper trapezius.

**Fig. 10:**
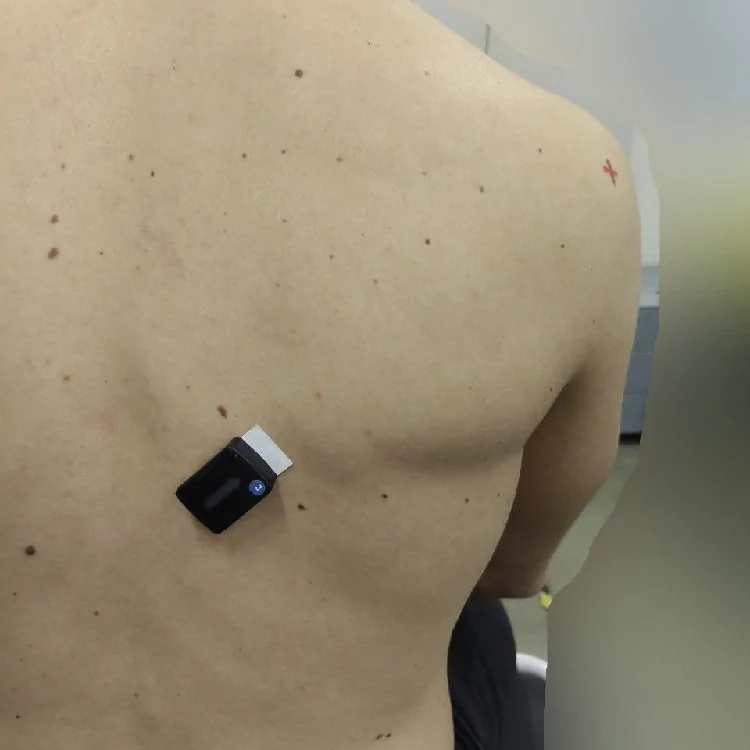
Sensor placement - lower trapezius.

**Fig. 11:**
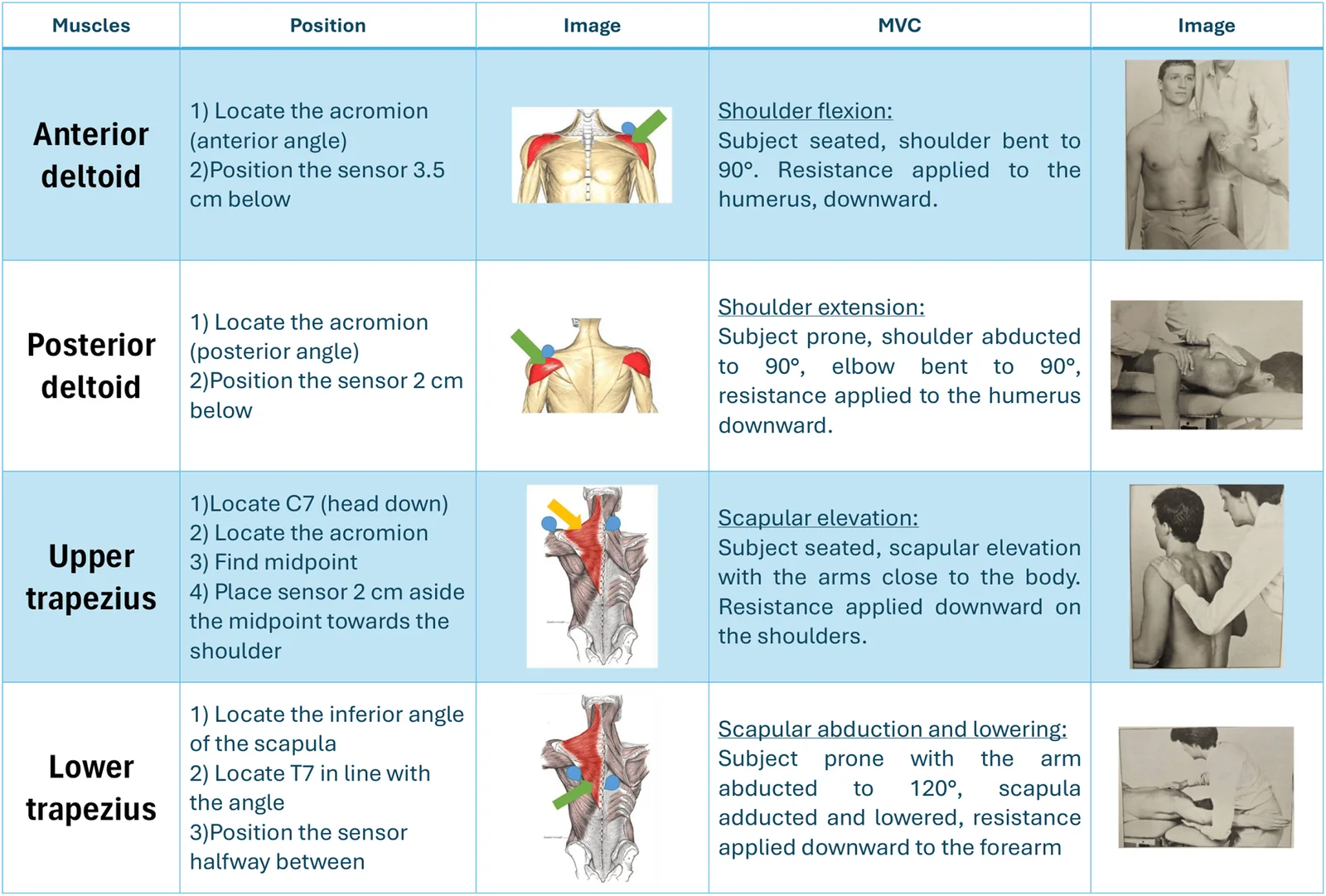
Contents of sensor_placement_and_mvc.pdf. The Position column provides instructions for locating the target muscles and placing the EMG sensors, while the MVC column describes the procedures for recording the MVC of each muscle. The corresponding images illustrate sensor placement and MVC acquisition, respectively.

Finally, before data acquisition began, signal quality was assessed visually using a custom Python interface developed specifically for this purpose and built on the official Delsys Python API (version 2.8.0). The application was run on a computer using Windows 11 and was used to:

• configure the sensor parameters
• navigate through the experimental procedure
• trigger signal acquisition
• label the acquired signals

At the end of the recording session, the custom application was also used to organise and store the acquired data.

### 4.3 Experimental procedure

After the quality of the EMG signals had been assessed, the setup was complete and the experimental session began. The recording sequence was as follows:

1. Signal at rest
2. Shoulder abduction – 10 repetitions
3. Shoulder abduction – 30 s
4. Shoulder flexion – 10 repetitions
5. Shoulder flexion – 30 s
6. Rower – 10 repetitions
7. Rower – 30 s

During phase 1, the robot was not used, and EMG signals were acquired while participants were seated upright at rest.

Between phases 1 and 2, an MVC signal was recorded for each muscle in the following sequence:

1) Rear deltoid
2) Lower trapezius
3) Anterior deltoid
4) Upper trapezius

The procedures used to elicit maximum force from each muscle are described in *sensor_placement_and_mvc.pdf* (Fig. 11) and were established in consultation with an expert physiotherapist. An example of MVC recording during a single experimental session is shown in Fig. 12, Fig. 13, Fig. 14, Fig. 15.

**Fig. 12:**
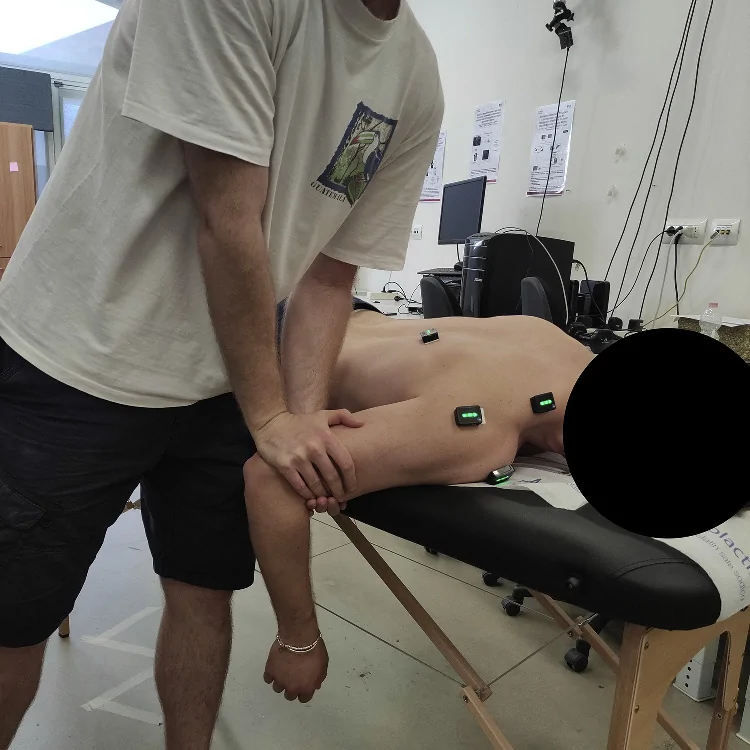
MVC recording - rear deltoid.

**Fig. 13:**
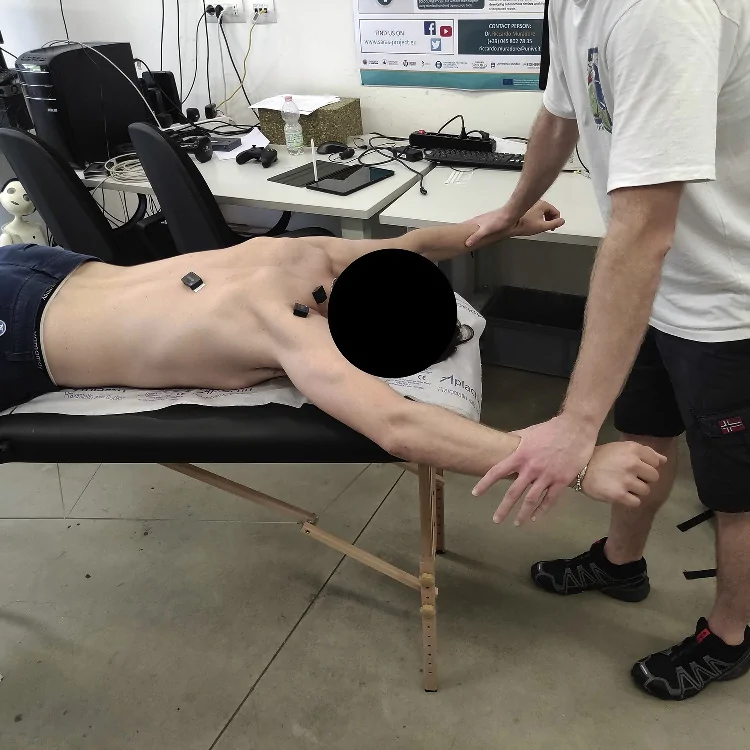
MVC recording - lower trapezius.

**Fig. 14:**
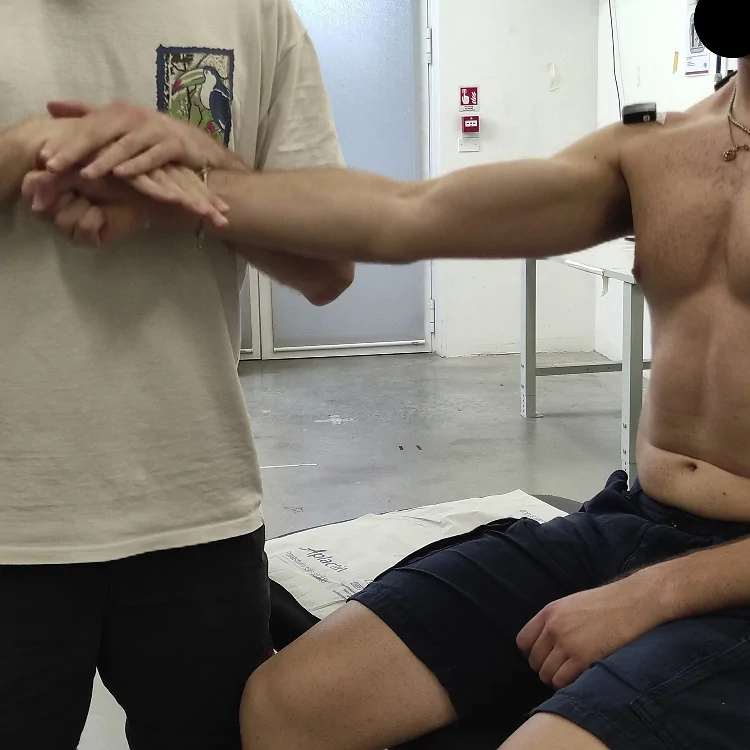
MVC recording - anterior deltoid.

**Fig. 15:**
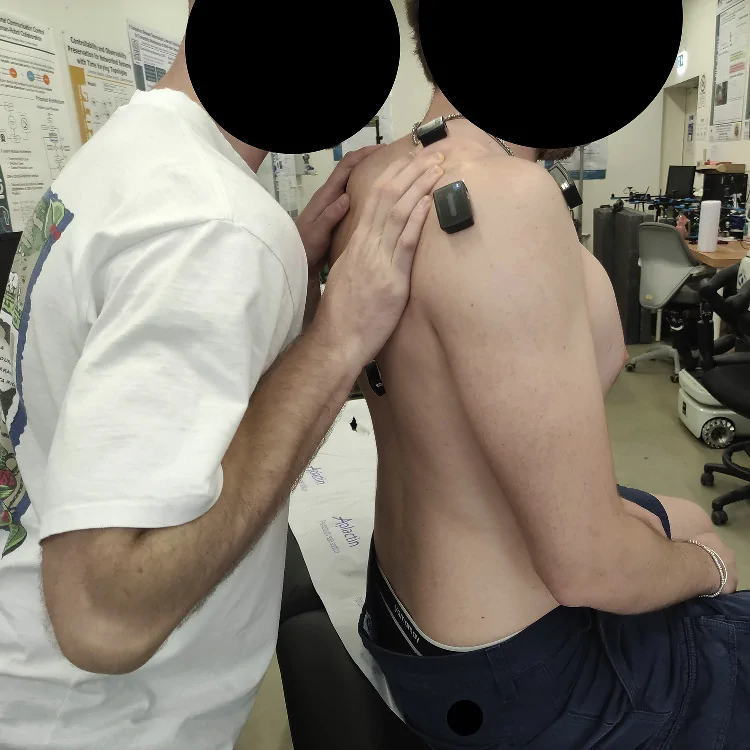
MVC recording - upper trapezius.

After MVC recording and before phase 2, the path constraining the robot during shoulder abduction and flexion was adjusted using the control method described in [17]. The path was based on each participant’s upper-limb length and functional movements. With the elbow extended, the distal end of the limb follows a circular path during shoulder abduction and flexion [18,19]. A circle was therefore used to constrain the robot’s motion. To define it, the Cartesian positions of the robot end effector were recorded in the following sequence (Fig. 16, Fig. 17, Fig. 18):

1. The handle was placed next to the participant’s shoulder; this point was defined as the centre of the work circle (**C**).
2. The participant held the handle with the arm and wrist in neutral positions; this point defined the circle radius ***r*** and was used to compute the initial unit vector **a**.
3. The participant held the handle at 90° in front of the body along the sagittal plane; this point was used to obtain a second unit vector, **v1**.

**Fig. 16:**
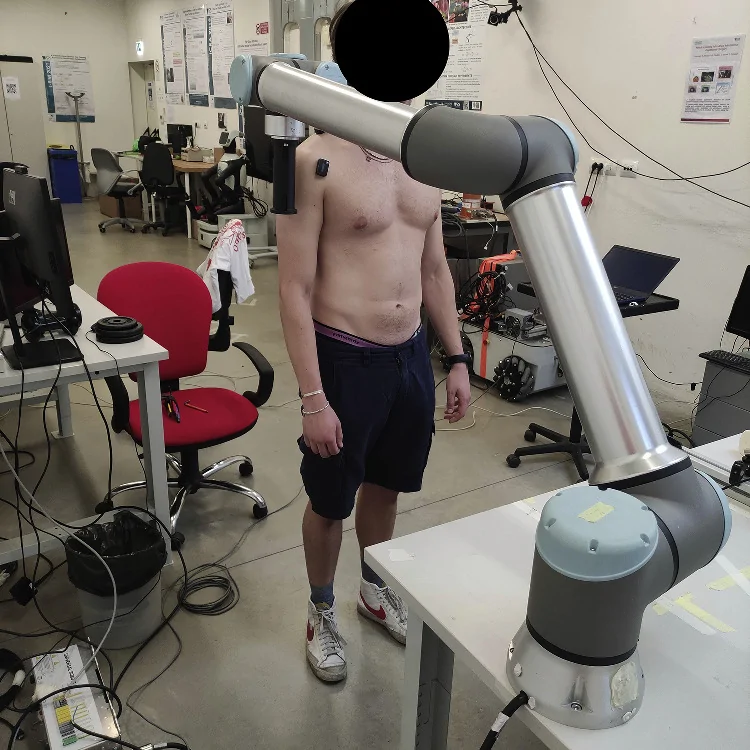
Circle path setup - step 1.

**Fig. 17:**
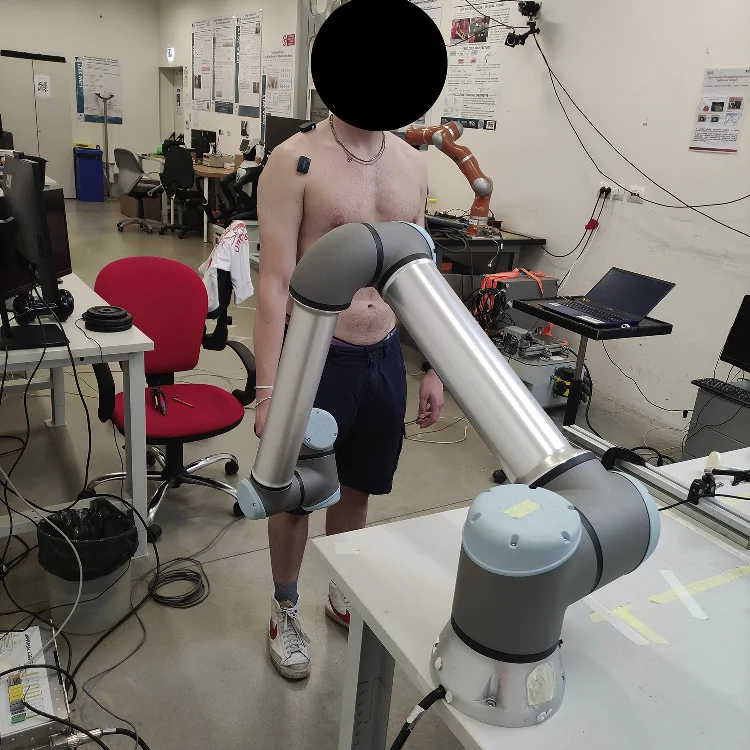
Circle path setup - step 2.

**Fig. 18:**
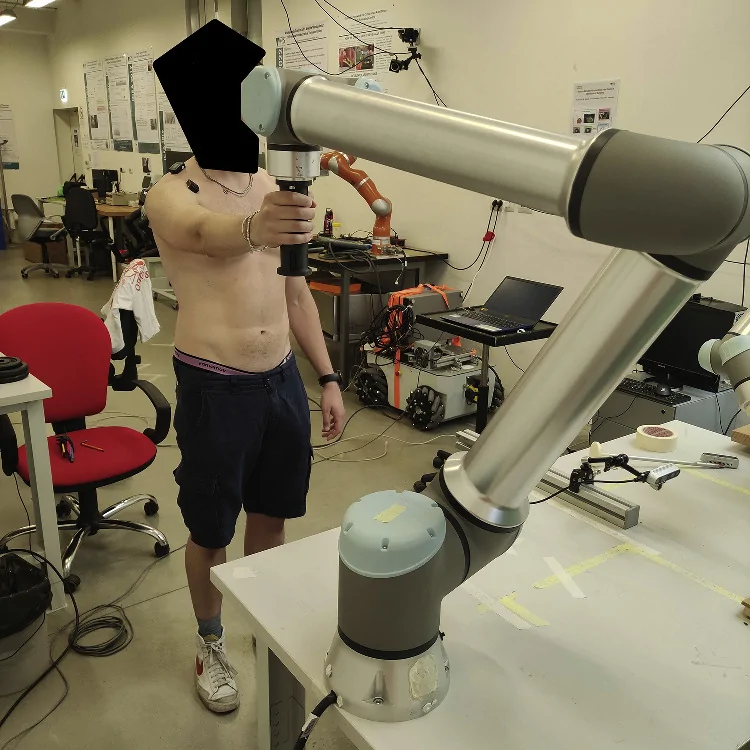
Circle path setup - step 3.

After the 3D coordinates had been recorded, vector **b**, orthogonal to **a** and lying in the same plane, was computed using a double vector product:

a×v1=v2

v2×a=b

Finally, a fixed number of points on circle **y** were obtained by interpolating its parametric equation, defined as:

y=C+rcos(θ)a+rsin(θ)b

The robot controller and reference-path generation were implemented using the ROS framework on a computer running Ubuntu 20.04, separate from the computer running the custom Python application.

Finally, between phases 5 and 6, the reference path used to constrain the robot during the rower exercise was recorded as follows:

1. The participant held the handle with the upper arm and forearm at a 90° angle in the sagittal plane (Fig. 19).

**Fig. 19:**
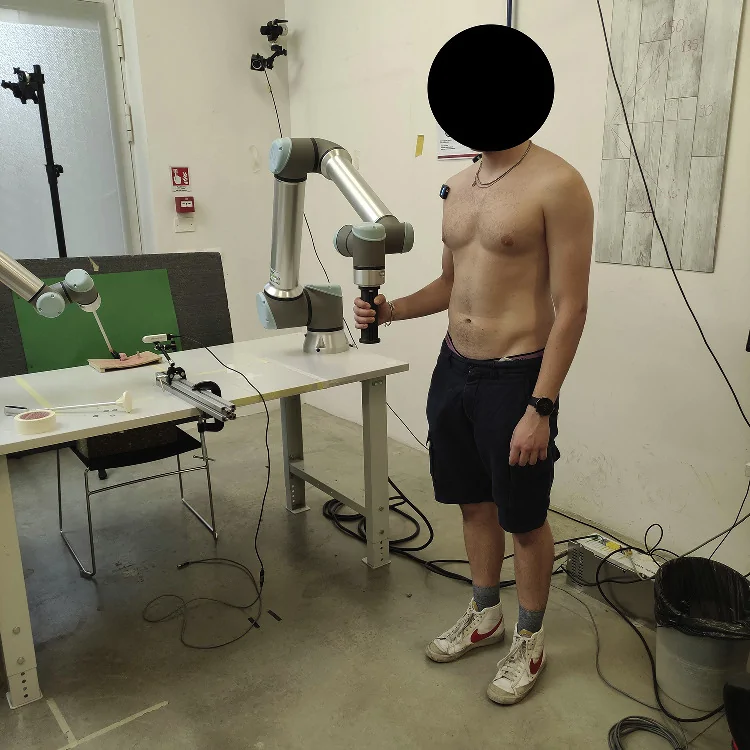
Row path setup - step 1.
2. The first spline point was recorded with the handle in an “over-reach” position, at the same height above the ground as the starting position. The arm was fully extended forward, and the torso was slightly rotated to increase reach (Fig. 20).

**Fig. 20:**
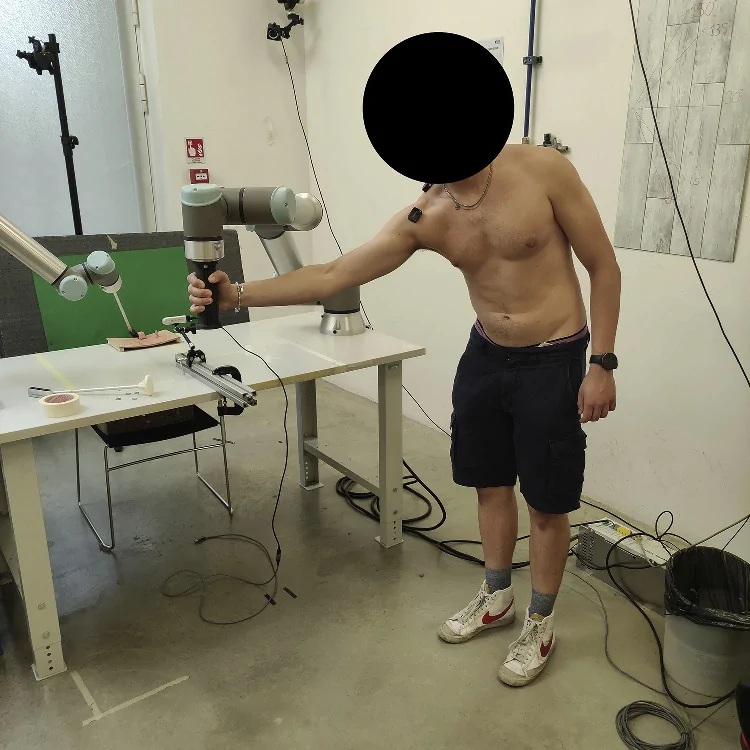
Row path setup - step 2.
3. The second and final spline point was recorded with the handle in an exaggerated scapular-retraction position at the same height above the ground as the starting position (Fig. 21).

**Fig. 21:**
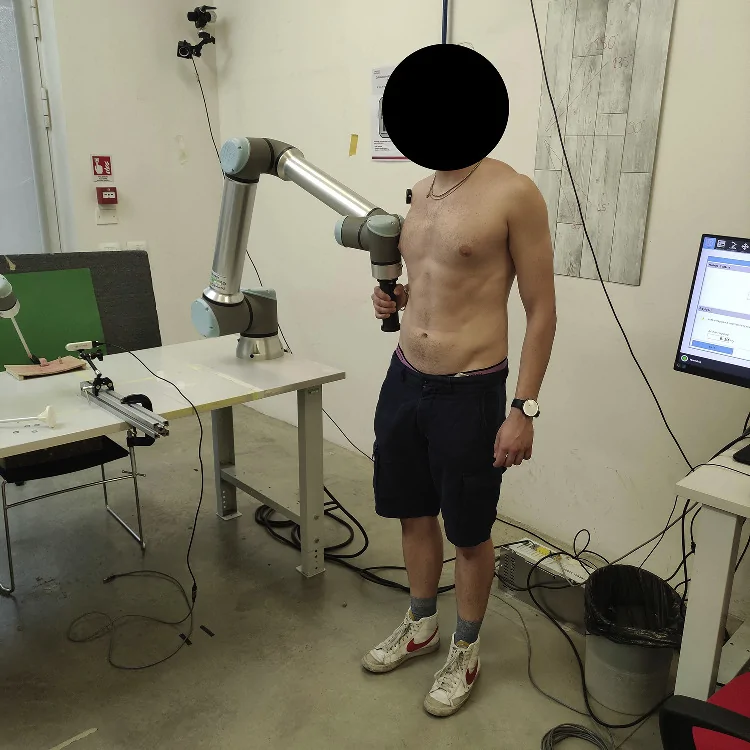
Row path setup - step 3.

This procedure ensured that the rower movement did not reach the endpoints of the curve recorded in steps 2 and 3 above. Fig. 22, Fig. 23, Fig. 24, Fig. 25 show examples of one participant during the exercise phases.

**Fig. 22:**
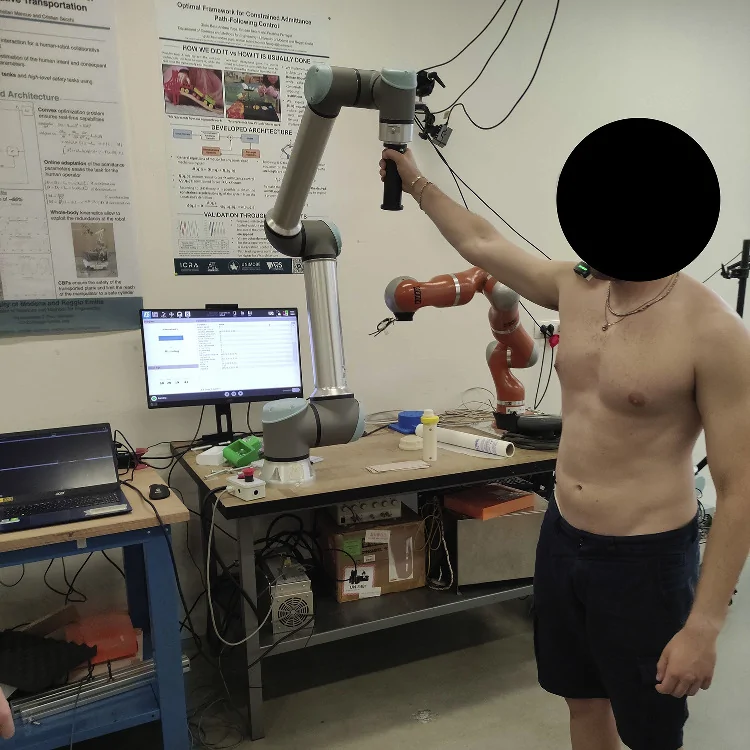
Shoulder abduction exercise.

**Fig. 23:**
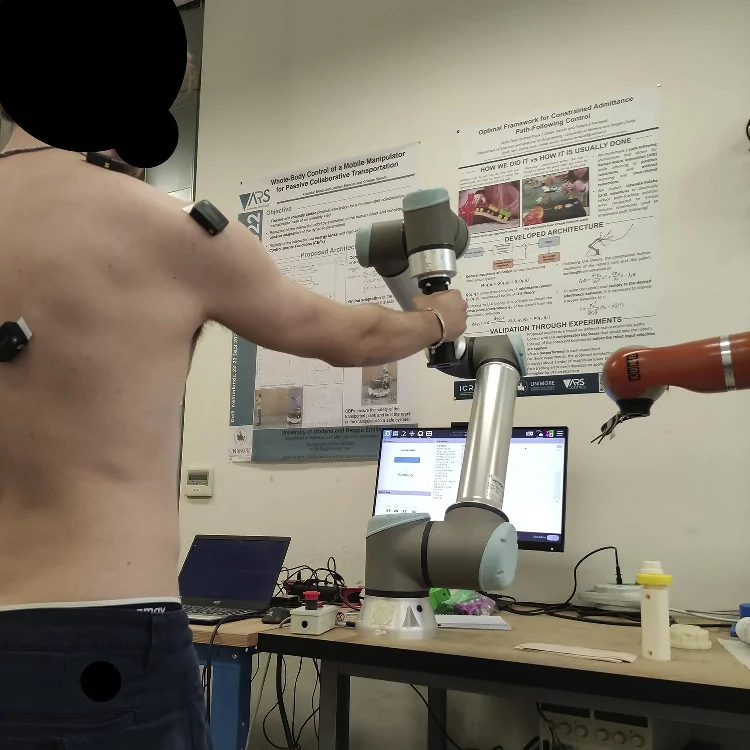
Shoulder flexion exercise.

**Fig. 24:**
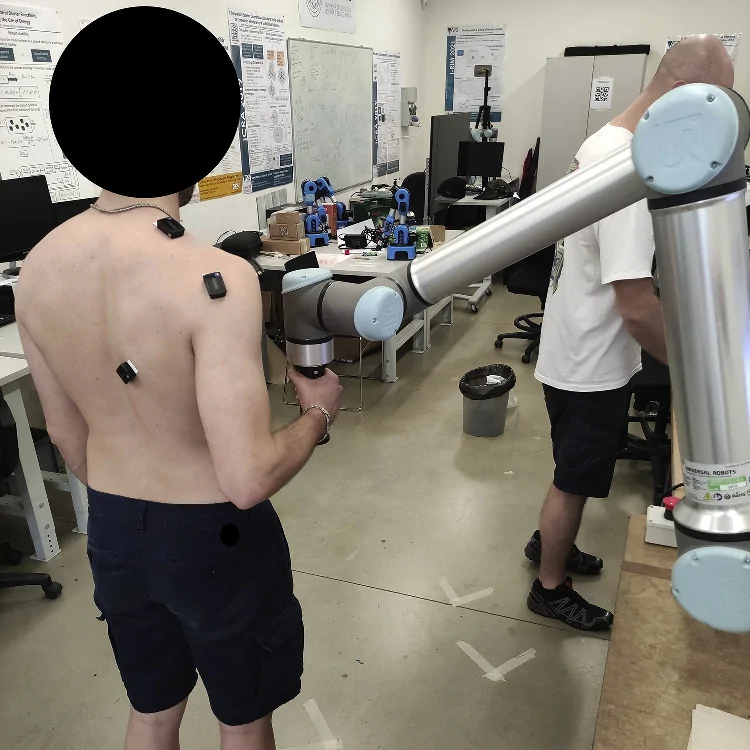
Row exercise (elbow flexion)**.**

**Fig. 25:**
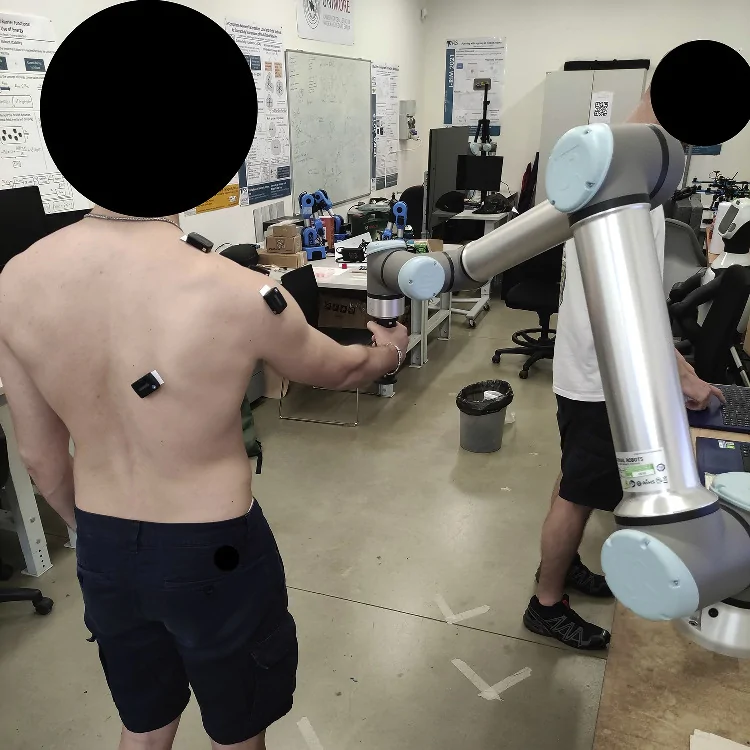
Row exercise (elbow extension),.

### 4.4 PAIN behaviours simulation AND labelling process

After the work-circle path had been tuned and before phase 2 began, participants were instructed to reproduce three behaviours simulating pain occurrences. These behaviours were selected based on [12] and in consultation with an expert physiotherapist:

• **Upper trapezius compensation**: incorrect activation of the upper trapezius on the active-limb side by raising the shoulder towards the ear. This muscle is generally monitored to assess compensatory activity because a healthy participant would not normally activate it in this compensatory manner during these exercises.
• **Limb sudden stiffness**: the active limb was suddenly stiffened during movement; in the EMG signal, this behaviour resembles a sudden pain occurrence.
• **Hesitation**: interruption of limb movement, followed by a slow resumption until the normal movement velocity was restored. This behaviour represents a situation in which a patient anticipates approaching a painful configuration; anticipation of pain is generally assessed in such instances.

Participants simulated these behaviours while performing the three orthopaedic rehabilitation exercises described above: shoulder abduction, shoulder flexion, and rower. All movements were performed using the robot. During each exercise, participants waited for an oral cue from the researcher. Upon receiving the cue, they simulated one of the pain-related behaviours. No constraint was placed on which behaviour to reproduce; participants selected one freely each time the cue was given. Simultaneously, the researcher used the custom Python application to label the signal in real time.

It should be noted that these labels do not correspond exactly to the start and end times of the simulated behaviour. This discrepancy resulted directly from the labelling procedure: the positive (i.e. pain-related) label began when the researcher pressed and held a button while giving the participant an oral cue, and ended when the button was released. Consequently, the entire simulation period was labelled as positive, although some uncertainty remained at the beginning and end of the labelled signal interval.

At the end of the recording session, the acquired data were stored in the participant’s folder within *Raw_Data*. To generate the data in the corresponding participant folder within *Final_Dataset*, the raw data were automatically processed using the custom Python application.

## Limitations

The dataset was collected from a homogeneous population of Italian adults aged 21–41 years; therefore, the recordings may not represent the general population, particularly younger and older individuals. Moreover, data were acquired from healthy participants who simulated pain-related movements; consequently, the dataset may lack the consistency and reliability required for clinical research. The experimental design included only three specific movements, which do not cover all shoulder-rehabilitation exercises and may limit generalisability to other shoulder movements. In addition, the specific simulated pain-related behaviour was not labelled, resulting in a purely binary labelling strategy. Muscle fatigue may also be more prevalent in the rower EMG signals because this was the final exercise performed. Finally, the data were recorded under controlled laboratory conditions and therefore do not capture the variability present in real clinical environments.

## Ethics Statement

The study was conducted in accordance with the principles of the Declaration of Helsinki. All participants were fully informed about the experimental procedure and provided written informed consent before participation. The experimental procedure was reviewed and approved by the Research Ethics Committee of the University of Modena and Reggio Emilia (Reggio Emilia, Emilia-Romagna, Italy) under protocol number 321,787/2025. A copy of the informed-consent form is provided with the submission.

## Credit Author Statement

**Giulio Besi:** Conceptualization, Methodology, Validation, Investigation, Supervision, Resources, Data curation, Formal analysis, Software, Writing – Original Draft, Writing – Review & Editing; **Alessandra Fava:** Resources, Data curation, Writing – Original Draft; **Haoxiang Wu:** Software, Resources; **Sara Garavini:** Methodology, Resources; **Lorenzo Sabattini:** Supervision, Writing – Review & Editing; **Federica Ferraguti:** Supervision, Project administration, Writing – Review & Editing.

## Declaration of Generative AI and AI-Assisted Technologies in the Manuscript Preparation Process

During the preparation of this work, the author(s) used ChatGPT (OpenAI) for shortening the text length and improving readability. The author(s) reviewed and edited the output as needed and take full responsibility for the content of the published article.

## Data Availability

ZenodoSimulated Pain in Robotic Orthopaedic Shoulder Rehabilitation (Original data).
